# Energy efficient relay selection framework for 5G communication using cognitive radio networks

**DOI:** 10.1038/s41598-025-00068-5

**Published:** 2025-05-04

**Authors:** S. Esakki Rajavel, Stalin Allwin Devaraj, A. Andrew Roobert, Om Prakash Kumar, Shweta Vincent

**Affiliations:** 1 Electronics and Communication Engineering, Faculty of Engineering, Karpagam Academy of Higher Education, Coimbatore, Tamil Nadu 641021 India; 2 Electronics and Communication Engineering, Francis Xavier Engineering College, Tirunelveli, Tamil Nadu 627003 India; 3 Electronics and Communication Engineering, Francis Xavier Engineering College, Tirunelveli, Tamil Nadu 627003 India; 4 Department of Electronics and Communication Engineering, Manipal Institute of Technology, Manipal Academy of Higher Education, Manipal, 576104 India; 5 Department of Mechatronics, Manipal Institute of Technology, Manipal Academy of Higher Education, Manipal, 576104 India

**Keywords:** Energy-efficient relay selection (EERS), Cooperative spectrum sensing, Cognitive radio network, 5G communication, SDG 7 (Affordable and clean energy), SDG 9 (Industry, innovation, and infrastructure), SDG 13 (Climate action), Engineering, Electrical and electronic engineering

## Abstract

The large bandwidth of 5G wireless networks results in a discontinuous optimal spectrum. This study leverages cognitive radio networks and collaborative spectrum sensing to improve the transmission performance in 5G communication. Energy limitations for each secondary user (SU) and potential errors in secondary transmission within cognitive nodes during cooperative transmissions and spectrum sensing contribute to the dynamic energy efficiency. This paper details an Electronic Energy Relay Selection (EERS) system. The weighted average function determines the optimal relays when the network communication power consumption and spectrum-detection levels are equal. The EERS system examines the correlation between energy efficiency and detection precision. The proposed EERS system surpasses the performance of the compressed sensing collaborative detection (CSCD) system. MATLAB was used to evaluate and compare performance metrics such as weighted energy consumption, number of collaborative SU relays, and probability of missing detection with those of compressed sensing-based collaborative detection.

## Introduction

Owing to applications that require a high amount of bandwidth, 5G connectivity is urgently needed. The demands for the spectrum and its available bandwidth are inversely correlated. Collaborative spectrum detection enhances the spectrum efficiency in Cognitive Radio (CR) networks^1,2^. A smart radio called a CR uses information from alerting nearby locations to choose how it will transmit. Ideally, the CR should be able to draw lessons from prior actions and experiences. Using an unlicensed spectrum reduces the issue of underutilising the permitted spectrum band^3–5^.

Researchers have examined several convex optimization issues to minimize the objective function in CRNs. To maximize the benefits of cooperative networks, Yao-Win Hong devised a spatial diversity system for multiuser wireless devices and introduced different power allocation techniques. The energy consumption challenges of cooperative spectrum sensing and spectrum sharing in energy-limited CRNs were examined in^6–8^. Collaborative spectrum sensing increases energy consumption while improving the recognition accuracy and lowering the objective function. Cooperative spectrum sharing based on green communication lowers the overall energy usage. For efficient data transfer, the linearization method can reduce the time and energy required for spectral detection^9–11^.

Literature^12^ investigates the issue of spectrum scarcity and the use of MAC protocols for current transmissions in cognitive radio users. Cooperative cognitive radio networks based on single and multiple relays have also been studied^13^. As mentioned above, recent research has examined energy-efficient cognitive radio networks. A high-energy economy is required in the network design of energy-restricted applications, such as wireless sensors and ad hoc sensor networks^14,15^.

Literature^16^ discusses the Efficiency of Upcoming Spectrum and the Detection of Primary Users. A Heterogeneous D2D Cognitive Radio Network focuses on spectrum lack problems. Different physical layer security has been introduced to enhance the safety of 5G methodologies^17,18^.

Relay range and power distribution for multi-basis collaborative Device2 broadcasts reduce communication interference^19^. To protect the data broadcast, an energy-harvested relay has been introduced^19,20^.

Literature^21^ discusses the Energy consumption and improvement of the network lifetime of 5G Relay Networks. To improve the Energy Efficiency of Relay Networks, an energy incentive model has been introduced^22^.

To improve system implementation under energy constraints, numerous research initiatives have been undertaken to designate accessible power assets effectively. However, it is possible that these arrangements are not directly applicable to cognitive radio networks.

Therefore, creative techniques for energy-efficient tactics are crucial in cognitive radio networks. This affirms that the collaborative relay methodology is a real method for constructing secondary broadcast and spectrum sensing presentations, has the potential for energy-efficient cognitive radio network designs, and can efficiently sense and utilize spectrum gaps.

This paper proposes an energy-efficient relay selection (EERS) system to reduce the networking and spectrum sensing levels of energy usage. The quadratic collaborative bit error limit and weighted average function were employed in false detection to determine the number of relays. The contributions to 5G communication are described below based on the above goals:

(i) Energy-Efficient Relay Selection (EERS) was proposed to reduce energy use in spectrum sensors and networks.
(ii) A weighted neutral function was devised to determine the number of ideal relays within the constraints of universal misdetection conditions and secondary collaborative bit error rate by explicit modulation systems.
(iii) Research has been conducted on how the energy efficiency and detection accuracy of the EERS system interact.

The remainder of this paper is organized as follows. The Energy Efficient Relay Selection (EERS) system is described in “Energy efficient relay selection (EERS) system” section. “Simulation results” section presents simulation results and comments. Finally, “Conclusion” section concludes the study.

## Energy efficient relay selection (EERS) system

**Fig. 1:**
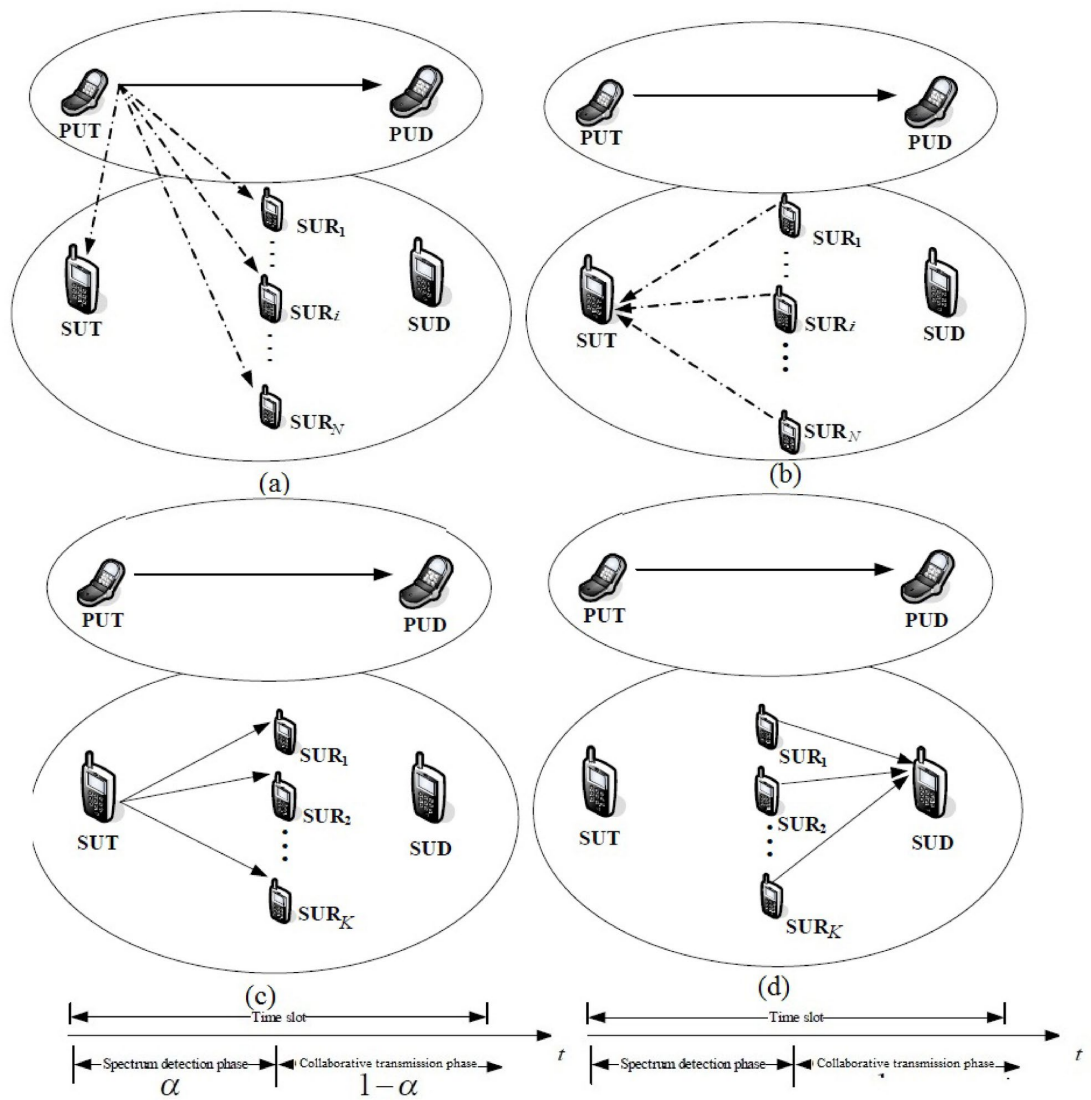
CR framework for 5G communication.

The network model for the EERS proposal is shown in Fig. 1. The CR framework includes EERS energy analysis and coordinated spectrum determination. Several SU Relays are available to support the secondary communication and spectrum sensing levels gathered by SU Transmitters, and several Secondary User Relays (SU Relays) are available. The SU relays assist the SU transmitter in identifying vacant spectra through cooperative spectrum detection. After reporting the local results to the SU transmitter for data union, the SU transmitter generates a global decision.

Based on the correctness of the data and the amount of energy expended during coordination and transmission, the SU transmitter selects the most effective SU relay. For coordinated communication via the EERS system, the SU transmitter was positioned at the top of the SU relay. This lowers the weight of the power consumption represented in the loss detection and serves as an example of the dependent bit error rate limit. Data are forwarded to the user’s Primary Device (PUD) through the Primary User Transmitter (PUT); however, when the SU transmitter and SU repeatedly identify an idle device, they report the PU Transmitter’s movement. The complete energy for the SU transmitter and relay is retained.

Numerous SU relays provide spectrum recognition and data transmission levels for the SU transmitters. N SU relays help the SU transmitter observe spectrum bands and spot communication opportunities at the initial sublevel (Fig. 1a) when the primary user (PU) is quiet in some spectrum bands. They used N orthogonal broadcast stations to transmit their requirements to the SU transmitter. The SU transmitter produces a universal choice in the following sublevel by customizing a specific union structure to chain the results of constrained sensing and varieties a universal conclusion in the second sublevel (Fig. 1b).

In Fig. 1c, the SU transmitter selects the optimal K (1 ≤ K ≤ N) SU relays in the third sublevel while paying close attention to the identification accuracy and energy usage. Temporarily, a secondary communication bit error rate condition and the global probability of a missed detection threshold are considered simultaneously. To select an appropriate SU relay and disseminate the transmitted signal to these specific SU at this sub-level, the SU transmitter precisely monitors the EERS system.

The signal is then sent to the Secondary User Destination (SUD) via the supplied K SU auxiliary Relays (understand the ideal SUR numeral is K), which use the Maximum Ratio Coupling (MRC) formula to create a new low-level signal (Fig. 1d). Additionally, the EERS system-based collaborative communication with factor and spectrum sensing slide with factor, where there is a standard parameter in the range of 0 to 1.

### Collaborative spectrum decision

The collaborative spectrum detection system that explores collaborative variety is displayed at the first sublevel of several user collaborative sensing. Detecting and broadcasting stations were developed to identify Rayleigh flat fading stations. Each SU relay uses its own restricted spectrum exposure to obtain results to the SU transmitter quickly enough that everyone can see them.

The SU transmitter discontinues the primary user’s attendance by fusing the local choices. For simplicity, the formidable union with the OR rule is operational at the SU transmitter as the union standard and energy recognition is realized for local spectrum recognition in the first sub-level (Fig. 1a).

Energy detection is an effective local spectrum recognition technique for cognitive radio networks. Each SU relay completes power detection at the broadcast window’s secure bandwidth B, and time T; m = BT equals the time-bandwidth products. As a follow-up result showing the initial customer presence/absence, the power signal produced by the ith SU relay = {SURi |i = 1, 2, · · ·, N } can be printed.

1 $${Y}_{{pr}_{i}}=\left\{\begin{array}{c}\sum_{t=1}^{2m}{[{n}_{{pr}_{i}}\left(t\right)]}^{2} {H}_{0}\\ \sum_{t=1}^{2m}{[\sqrt{{P}_{PU}. {h}_{{pr}_{i}}{ x}_{{pr}_{i}}\left(t\right)}+{n}_{{pr}_{i}}\left(t\right)]}^{2 }{H}_{1}\end{array}\right.$$

Here ${x}_{{pr}_{i}}\left(t\right)$ and ${n}_{{pr}_{i}}\left(t\right)$ represents Primary User (PU) spread indication and Additive White Gaussian Noise (AWGN) through null mean and variance correspondingly. ${h}_{{pr}_{i}}$ is damping constant. This is the first user communication signal, and AWGN is the number of points and differences from the first user. The damping constant of the search channel up to the relay is SU, and the time-bandwidth product is m = BT. The ${H}_{0}$ and ${H}_{1}$ denote the existence and absence of a user element, represent and link it.

The established signal energy at the SU transmitter is expressed as follows:

2 $${Y}_{pt}=\left\{\begin{array}{c}\sum_{t=1}^{2m}{[{n}_{pt}\left(t\right)]}^{2 }{H}_{0}\\ \sum_{t=1}^{2m}[{\sqrt{{P}_{PU}{h}_{pt}{x}_{pt}\left(t\right)}+{n}_{pt}(t)]}^{2 }{ H}_{1}\end{array}\right.$$

The ${x}_{pt}\left(t\right) and$ ${n}_{pt}(t)$ represent the initial user communication signal and AWGN, respectively; the mean and variance are zero. The damping factor $({h}_{pt})$ of the search channel extends from the first user to the SU transmitter.

3 $$\text{P}{r}_{f\left(m,\lambda \right)}=\text{E}\left[\text{Pr}\left\{Y>\lambda |{H}_{0}\right\}\right]=Pr\left\{{x}_{2m}^{2}>\lambda \right\}=\frac{\Gamma \left(m,\frac{\lambda }{2}\right)}{\Gamma \left(m\right)}$$

4 $$\begin{aligned} \Pr _{{d\left( {m,\lambda ,\gamma } \right)}} = & E\left[ {\Pr \left\{ {Y > \lambda |H_{1} } \right\}} \right] = \Pr \left\{ {x_{{2m}}^{2} \left( {2\gamma } \right) > \lambda } \right\} = \exp \left( {\frac{\lambda }{2}} \right)\mathop \sum \limits_{{n = 0}}^{{m - 2}} \frac{1}{{n!}}\left( {\frac{\lambda }{2}} \right)^{n} \\ & \quad + \left( {1 + \frac{\gamma }{\gamma }} \right)^{{m - 1}} .~~\left[ {\exp ( - \frac{\lambda }{{2\left( {1 + \gamma } \right)}} - \exp \left( { - \frac{\lambda }{2}} \right)\mathop \sum \limits_{{n = 0}}^{{m - 2}} \frac{1}{{n!}}\left( {\frac{{\lambda \gamma }}{{2\left( {1 + \gamma } \right)}}} \right)^{n} } \right] \\ \end{aligned}$$

5 $$P{r}_{m(m,\lambda ,\gamma )}=1-P{r}_{d\left(m,\lambda ,\gamma \right)}$$

Here $\gamma$ is the established signal-to-noise ratio (SNR) at Secondary User Transmitter, ${x}_{2m}^{2}$ and ${x}_{2m}^{2}\left(2\gamma \right)$ denotes the essential and non-essential chi square circulation by 2 m grades of choice and a non-essentiality limit $2\gamma$, correspondingly.

Every SU relay forwards its local statement to the SU transmitter at the following sub-level: At the SU transmitter or relay, the limited sensing outcomes can be interconnected as binary decisions, denoted as di€ (0, 1). Specifically, ‘0’ denotes the appearance of the Primary User, whereas ‘1’ denotes the primary user’s existence. In the OR law, all single-bit results are merged at the SU transmitter. OR union legislation is common for cognitive nodes to contact the approved primary user spectrum, reducing the possibility of producing inferences to the primary user.

The purpose of the OR union law is expressed as follows:

6 $${\text{Y}}_{\text{t}}=\sum_{i=1}^{N+1}{d}_{{\varvec{i}}}=\left\{\begin{array}{c}\ge 1 { H}_{1}\\ 0 {H}_{0}\end{array}\right.$$

The collaborative sensing sublevel is supported by N SU relays and a single SU transmitter. The entire spectrum sensing secondary user quantity is. It is critical to reflect on broadcasting channel errors that reduce the communication consistency of SU relay reporting outcomes for a union at collaborative recognition and reporting levels. Assuming that every cognitive node in the initial sub-level has a similar resident False Alarm Probability (${P}_{{r}_{f}}$) and resident Missed Detection Probability (${P}_{{r}_{m}}$), the universal FAP ${Q}_{f}\left(N\right)$ and MDP ${Q}_{m}\left(N\right)$ with the OR union law can be stated as

7 $${Q}_{f}\left(N\right)=1-\prod_{i=1}^{N+1}\left(\left(1-{P}_{{r}_{f}}\right)\left(1-{P}_{{r}_{e}}\right)+{P}_{{r}_{f}}{P}_{{r}_{e}}\right)$$

8 $${Q}_{m}\left(N\right)=\prod_{i=1}^{N+1}(P{r}_{m}(1-P{r}_{e})+\left(1-P{r}_{m}\right)P{r}_{e})$$

where Pr_e_ denotes the chance of inaccuracy in the broadcasting channels. Because the recognizing and broadcasting channels are IID for each SU relay, we may assume that each SU relay has a comparable detection probability locally, and the error rate of each broadcasting channel is similar. The universal probability of a missed detection can be expressed as follows:

9 $${Q}_{m}\left(N\right)={(P{r}_{m}\left(1-P{r}_{e}\right)+\left(1-P{r}_{m}\right)P{r}_{e})}^{(N+1)}$$

### EERS energy analysis

Energy usage at the information and communication level was also considered. As previously stated, the SU transmitter and each SU relay a specific energy when recognizing the primary user signals. However, awareness of electricity consumption is lower than that of media and communication networks. Consequently, the initial lower-level power consumption is ignored.

The first emphasis is on the energy expended by N SU relays to broadcast their recognition results to the SU transmitter in the next sublevel, representing the energy ingestion at the spectrum recognition level.

Consider that each SU relay generates a binary result based on its native comment and transmits the single-bit choice result to the SU transmitter for the union. Assume that single-bit broadcast data are moderated using BPSK, and the broadcast error rate is denoted as Pr_e_.

In the fault map, the Chernoff limit, which allows dual-phase shift switching with N actual transmit antennas (cooperative SU relay), is represented as:

10 $$P{r}_{e}\le {\left(\frac{{E}_{b}}{N.{N}_{0}}\right)}^{-N}$$

In the case of the signal-to-noise ratio, E_b_ represents the average communication power of one bit of the broadcast population in determining the outcome of each SU relay associated with the bit error of the broadcast channel Pr_e_. n is the exponent of wireless path loss factor. As a result, the following equation gives the highest limit of power consumption, which is evaluated as power consumption divided by cooperation level.

11 $${E}_{sensing}\le G\left(\frac{N.{N}_{0}}{{P{r}_{e}}^\frac{1}{N}}\right){d}_{sensing}^{n}$$

where d_sensing_ is the broadcasting distance between the SU relays and SU transmitter, which is less than d_transmission_ from the collaborating SU relays to the SU destination. To optimize the gain and hardware performance, the link line should have a constant G = 5 × 10^8^, connected to the continuous effect of the antenna, and n is a set of wireless networks. As a result, Eq. (11) indicates the power consumption of the power amplifier at the spectrum definition level.

Simultaneously, each SU relay and transmitter employs local information and binary category selection to limit the principal user’s presence/absence, and the power consumption of this low level is smaller than that of the cooperative level. At the guaranteed level, electricity consumption is normally equal to the received power and there is no guarantee or advertising rate limit for this level.

The power supply at the collaboration level mostly consists of the power supply P_PA_ and the circuit breaker utilizing the P_C_ in the cooperative SU relay in the communication network paper. At the collaborative communication level, the total energy usage for communicating a single bit can be approximated as:

12 $$E_{transmission} = \frac{{\left( {P_{C} + P_{PA} } \right)}}{{R_{b} }}\quad Joules$$

where ${R}_{b}$ represents the secondary communication rate is defined as for the sake of this study, let us assume that it is crucial to understand the bit equation R_b_ = R_s_ log2Mbit/s, where M stands for M-ary four. Amplitude modulation order or M-ary–M-ary phase-shift switch control to validate the communication QoS criteria. On the other hand, for wireless power batteries, the power consumption of communication power Pc_t_ and storage power Pc_r_ for each talent is roughly 97.3 mW and 111.4 mW, respectively.

Before selecting the best SU relays using the EERS approach, the SU transmitter includes all SU relays as N + 1 in the potential number of secondary users for a collaborative connection.

At different levels of communication, the SU Transmitter sends its signal to all SU Relays that it can find, and SU Relays aggregate in the sub-level of collaborative communication.

The power usage of the PC, which was formerly a fully functional generator, can be expressed as PC = (N + 1) (Pc_t_ + Pc_r_). On the other hand, the SU relay broadcast sublevel and the SU relay cooperative communication sublevel may experience channel fading. Consequently, the following is an analysis of the power amplifier balancing utilizing the PPA in the second communication.

13 $$E_{PA} = \frac{{P_{PA} }}{{R_{b} }} = GE_{b} d_{transmission}^{n} \quad {\text{Joules}}$$

Here, E_b_ is the average bit error power transmitted per bit during the second communication. The link border constant, G = 5 × 10^8^, is the same as the factor shown. Recognize that d_transmission_, which is much greater than d_sensing_ and the distances between collaborative SU relays, represents the distance between the collaborative SU relay set and the SU destination. In the case of n = 2 empty spaces and typically n = 3 for fading channels, n is the exponent of wireless path fading. For collaborative communications, the total energy consumption per bit can be expressed as

14 $$E_{transmission} = \frac{{\left[ {\left( {N + 1} \right)\left( {P_{ct} + P_{cr} } \right)} \right]}}{{R_{b} }} + GE_{b} d_{transmission}^{n} \quad J$$

The quantity of handshakes and d_transmission_ in the communication BER limit appear to be related to E_transmission_. Collaborative diversity and a sense of integrity can grow as the number of collaborations increases.

Consider the scenario in which each connection of the SU relay is estimated as an independent distribution (IID), and the transmission signal is regulated by M-ary phase-shift switching or M-ary quadrature amplitude modulation. The SU destination associates the received signal using the maximum ratio combining standard, considering that each collaboration node has similar communication power. Consequently, we can now demonstrate the normalized SNR of the SU word.

15 $$\gamma_{b} = \frac{{\left| {\left| H \right|} \right|_{F}^{2} }}{N + 1}\frac{{E_{b} }}{{N_{0} }}\quad dB$$

The distribution of transmit power to the SU transmitter and the N number of SU relays during the coordination phase are included in the denominator (N + 1). The power spectral density of N_0_ is entirely Gaussian. This represents the collaborative communication channel matrix model developed by Frobenius. The origin of the channel matrix is a circularly symmetric complex Gaussian variable with mean and variance of zero. If every association exhibits IID flat fading, ${||H||}_{F}^{2}=\sum_{i=1}^{2(N+1)}{|{h}_{i}|}^{2}$ follow the average chi-square distribution, for instance, with two (N + 1) degrees of freedom ${X}_{2(N+1)}^{2}$. The probability density function (PDF) of F can be derived as follows:${f}_{{\left|\left|H\right|\right|}_{n}^{2}}\left(x\right)=\frac{1}{N!}{x}^{N}{e}^{-x},x\ge 0$ F.

As a result, the BER is indicated as follows for SU targets employing multiphase-shift keying or Mult quaternary amplitude modulation:

16 $$P{r}_{b}\left(N,{\gamma }_{b}\right)={\int }_{0}^{\infty }P{r}_{b,AWGN({\gamma }_{b}){f}_{{\left|\left|H\right|\right|}_{F}^{2}}(x)}dx$$

where Pr_b_, the total white Gaussian noise, is the sum of the bit error rate in one additional channel of white Gaussian noise and the received signal-to-noise ratio of the M-ary phase shift switch from M-ary quaternary amplitude modulation. while maintaining with ${P}_{{r}_{b,AWGNMPSK}({\gamma }_{b})}=\frac{2}{b}Q\left(\sqrt{2{b}_{\gamma b}{sin}^{2}\left(\frac{\pi }{M}\right)}\right)$ and $P{r}_{b,AWGNMQAM(\gamma b)}=\frac{4}{b}\left(1-\frac{1}{\sqrt{M}}\right)Q\left(\frac{\sqrt{3{b}_{\gamma b}}}{M-1}\right)$ to and represent the particular BER equations for the M-ary PSK and M-ary QAM modulation of AWGN channels, respectively, correspondingly $Q\left(x\right)=\frac{1}{\sqrt{2\pi }}{\int }_{x}^{+\infty }\text{exp}\left(-\frac{{t}^{2}}{2}\right)dt$ where the modulation order exponent is b = log2*M*.

By independently applying Pr_b_, AWGN MPSK (${\gamma }_{b}$), Pr_b_, additive white Gaussian noise MQAM (${\gamma }_{b}$), and related Eqs. (16), it is possible to show that Eq. (17) is a collaborative function of the steady projected signal-to-noise ratio and the SU relay amount. To obtain the integral formula below, we consider the integral table.

17 $${\int }_{0}^{+\infty }\begin{array}{c}{x}^{2n-1}{e}^{\frac{{-x}^{2}}{2}}Q\left(\frac{x}{\beta }\right)dx=\frac{\left(n-1\right)!}{2}{\left[1-{{(\beta }^{2}+1)}^{\frac{-1}{2}}\right]}^{n }.\\ \sum_{k=0}^{n-1}{2}^{-k}\left(\frac{n-1+k}{k}\right)[{1+\left({\beta }^{2}+{1)}^{\frac{-1}{2}}\right]}^{k}\end{array}$$

Set ${\beta }^{1}$ to be $\sqrt{\frac{\left(N+1\right)}{{b}_{\gamma b}{sin}^{2}\left(\frac{\pi }{M}\right)}}$ and n = *N* + 1 for M-ary PSK modulation. The closed-form equation of the bit error rate is expressed as

18 $$P{r}_{b,MPSK}\left(N,{\gamma }_{b}\right)=\frac{1}{{2}^{N}b}({1-\left({1+{\beta }^{2})}^{\frac{-1}{2}}\right)}^{N+1}$$

For M-ary QAM modulation, *n* = *N* + 1, and the closed-form equation of the bit error rate is expressed as

19 $$r_{{b,MQAM}} \left( {N,\gamma _{b} } \right) = \frac{{2\left( {1 - \frac{1}{{\sqrt M }}} \right)}}{{2^{N} b}}\left( {1 - \left( {1 + \beta ^{{2)}} } \right)^{{ - \frac{1}{2}}} } \right)^{{N + 1}} \cdot \sum\limits_{{k = 0}}^{N} {2^{{ - k}} } \left( {\frac{{N + k}}{k}} \right)\left( {1 + \left( {1 + \beta ^{2} } \right)^{{ - \frac{1}{2}}} } \right)^{k}$$

From the above equation, the Chernoff bond for the conventional BER of M-ary PSK with (N + 1) low-level antennas is shown as $P{r}_{b,MPSK}\left(N,{\gamma }_{b}\right)\le {\left(\frac{{E}_{b}}{\left(N+1\right){N}_{0}}\right)}^{-\left(N+1\right)}$ The Chernoff limit for the usual BER of M-ary QAM modulation with straightforward antennas is demonstrated to be:$P{r}_{b,MQAM}(N,{\gamma }_{b})\le \frac{4}{b}(1-\frac{1}{{2}^\frac{b}{2}})(\frac{1.5b{E}_{b}}{\left(N+1\right)\left({2}^{b}-1\right){N}_{0}}{)}^{-\left(N+1\right)}$ in the cooperative MISO system of the accordingly. The power required for MPSK modulation on one side can then be provided as a bit.

20 $${E}_{b,MPSK}\le \frac{(N+1){N}_{0}}{P{r}_{b,MPSK}^{\frac{1}{N+1}}(N,{\gamma }_{b})}$$

This is used to indicate a stable signal-to-noise ratio per bit. Additionally, we determine the upper bound of 1E_b_ for M-ary QAM modulation, expressed as

21 $${E}_{b,MQAM}\le \frac{2\left({2}^{b}-1\right)\left(N+1\right){N}_{0}}{3b}{\left(\frac{P{r}_{b,MQAM}}{\frac{4}{b}\left(1-\frac{1}{\sqrt{{2}^{b}}}\right)}\right)}^{\frac{-1}{N+1}}$$

The bound shown in Eq. (3.22) can be shortened and it can be said as

22 $${E}_{b,MQAM}\le \frac{2\left({2}^{b}-1\right)\left(N+1\right){N}_{0}}{3{b}^{\left(1+\frac{1}{N+1}\right)}}{\left(\frac{P{r}_{b,MQAM}}{4}\right)}^{\frac{-1}{N+1}}$$

Therefore, the suggested EERS system is restricted to the general MDP at the detection stage and the quadratic BER at the transmission stage, where the weighted average function is a function of all conceivable SU relay numbers N. For M-ary PSK and M-ary QAM modulation, respectively, we replace Eqs. (20) and (22) with Eq. (14) and use Eqs. (11) and (14) to generate a weighted Average E(N) that maximizes the variable N. This issue can be stated as follows:

23 $$\left\{ {\begin{array}{*{20}c} {\min E\left( N \right), } \\ {N Q_{m} \left( N \right) \le Q_{m,req,} } \\ {s.t. Pr_{b} \left( {N,\gamma_{b} } \right) \le Pr_{b,req,} } \\ \end{array} } \right.,\quad N > 0$$

where E(N) =$\alpha {E}_{sensing}+(1-\alpha ){E}_{transmission}$ represents the over-all energy consumption for single bit communication, which is built as a Weighted neutral purpose that signifies the weighted energy consumed in equally detection and communication phases. factor α is the standardized weight aspect in the choice of 0 < α < 1, which signifies band detection overhead in band recognition stage while (1 − α) is the standardized factor in collaborative communication stage.

24 $$E_{{MPSK}} \left( N \right) = \alpha G\left( {\frac{{N.N_{0} }}{{Pr_{e}^{{\frac{1}{N}}} }}} \right)d_{{sensing}}^{3} + \left( {1 + \alpha } \right)\left( {\frac{{\left[ {\left( {N + 1} \right)\left( {P_{{ct}} + P_{{cr}} } \right)} \right]}}{{R_{b} }} + G\frac{{\left( {N + 1} \right)N_{0} }}{{Pr_{{b,MPSK}}^{{\frac{1}{{N + 1}}}} }}d_{{sensing~}}^{3} } \right)$$

25 $${E}_{MQAM}\left(N\right)=\alpha G\left(\frac{N.{N}_{0}}{P{r}_{e}^\frac{1}{N}}\right){d}_{sensing}^{2}+\left(1+\alpha \right)\left(\frac{\left[\left(N+1\right)\left({P}_{ct}+{P}_{cr}\right)\right]}{{R}_{b}}+G{E}_{b,MQAM}{d}_{transmission}^{3}\right)$$

EERS is set up as tracks.

Stage 1: Collaborative sensing energy consumption research. Equation (11) uses the power consumption of the power supply of the low-level emission as the main power of the power spectrum, and the results in the field of each SU relay are purposefully modulated by the BPSK and sent to the SU transmitter for integration.

Stage 2: Research on collaborative communication energy usage Eq. (14), which describes the energy consumption at the collaborative communication level, also includes the power amplifier and circuit block energy consumption. Equation (20) assumes the upper bound of the regular necessary energy per bit if M-ary PSK modulates the conveyed signal. Equation (22) shows the upper bound of the regular essential energy per bit if the conveyed signal is modulated by M-ary QAM.

Stage 3: Challenging the invention of the EERS system. Make a weighted neutral function using a standardized weight, energy consumption formulation, and levels of sensing and communication. E(N) = αE_Sensing_ + (1 − α) E_transmission_, where α stands for the standardized spectrum sensing weight element and (1 − α) for the standardized collaborative communication weight element, is the representation of the weighted energy consumption neutral function for single-bit communication. E_sensing_ and E_transmission_ represent collaborative sensing and communication energy, respectively, known in Stages 1 and 2, respectively. The weighted energy consumption equation is formulated by Eq. (24) regarding M-ary PSK modulation. Equation (25) describes M-ary QAM modulation. Focusing on the universal MDP restriction at the sensing level and secondary BER restriction at the communication level is challenging when optimizing an EERS system. As a result, Eq. (23) specifies the optimization difficulty.

Stage 4: It is challenging to explain the EERS system optimization. It has been determined that the weighted neutral function and limitation settings are fully convex in the case of SU relay number N. The Non linear convex optimization is challenging for Eq. (23). Mathematical division is used to arrive at a conclusion after looking at the top digit K of the SU Relay set. The Karush–Kuhn–Tucker (KKT) notion is equivalent to the last point of E (K), which is the top collaborative SU relay set size if the last point of E (K) is the top solution. The EERS system is optimal if the KKT concept is a number. The KKT concept should then be refined to a number using the best SU relay passing technique. As a result, the EERS system achieves the best collaborative SU relay set by replicating the energy efficiency sideways by identifying precision and communication consistency, which helps to finish the production and key of a challenging nonlinear convex optimization problem.

## Simulation results

The simulation results and debates for the proposed EERS system are detailed in this section. An analysis was conducted on performance variables such as weighted energy consumption, Collaborative SU Relay Numbers, and Missing detection probability. The simulation variables used in the analysis of the EERS System are listed in Table 1.

**Table 1:** EERS simulation factors.

Factors	Settings
Time bandwidth product m	5
Average SNR range	0 to 20 dB
rate of reporting channel error P_re_	10–3/10–4
noticing the above	02./05/0.8
SU Relays N	79
Rs is the minimum transmission symbol rate	10KBaud
SNR at SU Destination ϒ_b_ range	5–20 dB
Collaborative communication distance d_transmission_ range	50–200 m
Reporting channel distance d_sensing_	5 m
Channel route loss factor n for fading	3
PSD AWGN	− 174 dBm

**Fig. 2:**
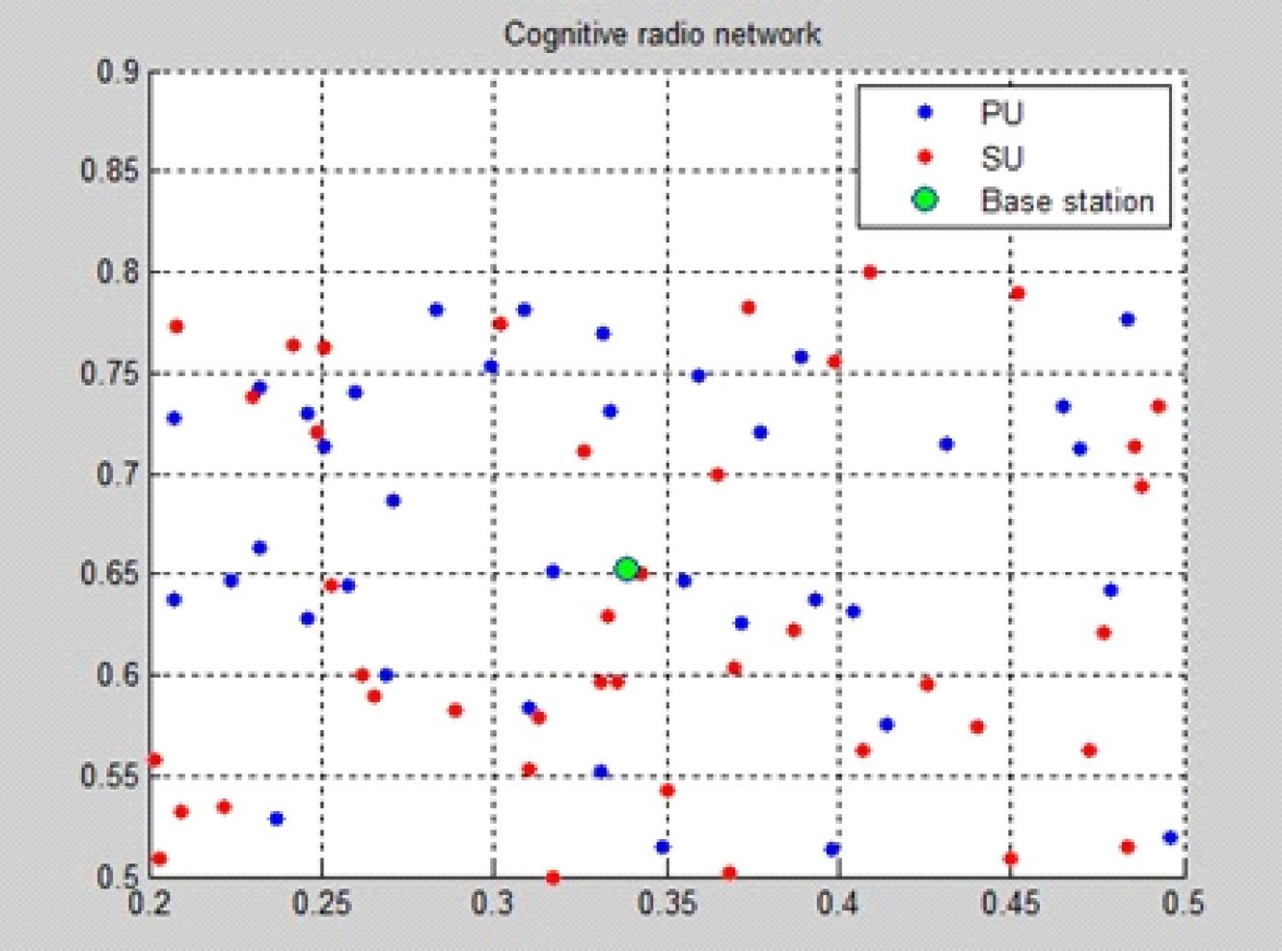
EERS network creation.

The EERS network construction scenario is illustrated in Fig. 2. The green dot indicates the EERS base station, the red dot is the secondary user, and the blue dot is the primary user of the system.

**Fig. 3:**
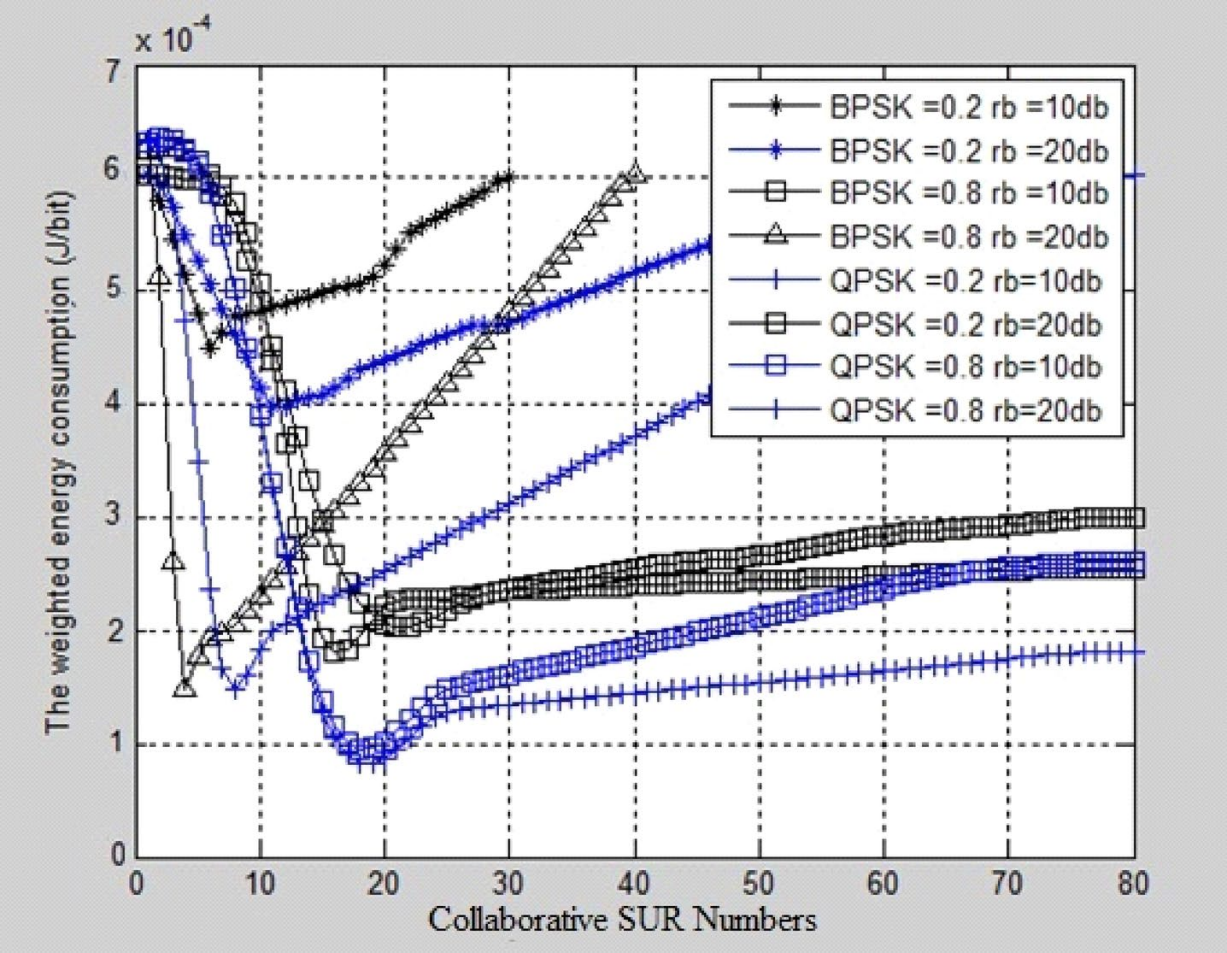
Weighted energy consumption comparison graphs vs collaborative SUR numbers at different SNR levels.

**Fig. 4:**
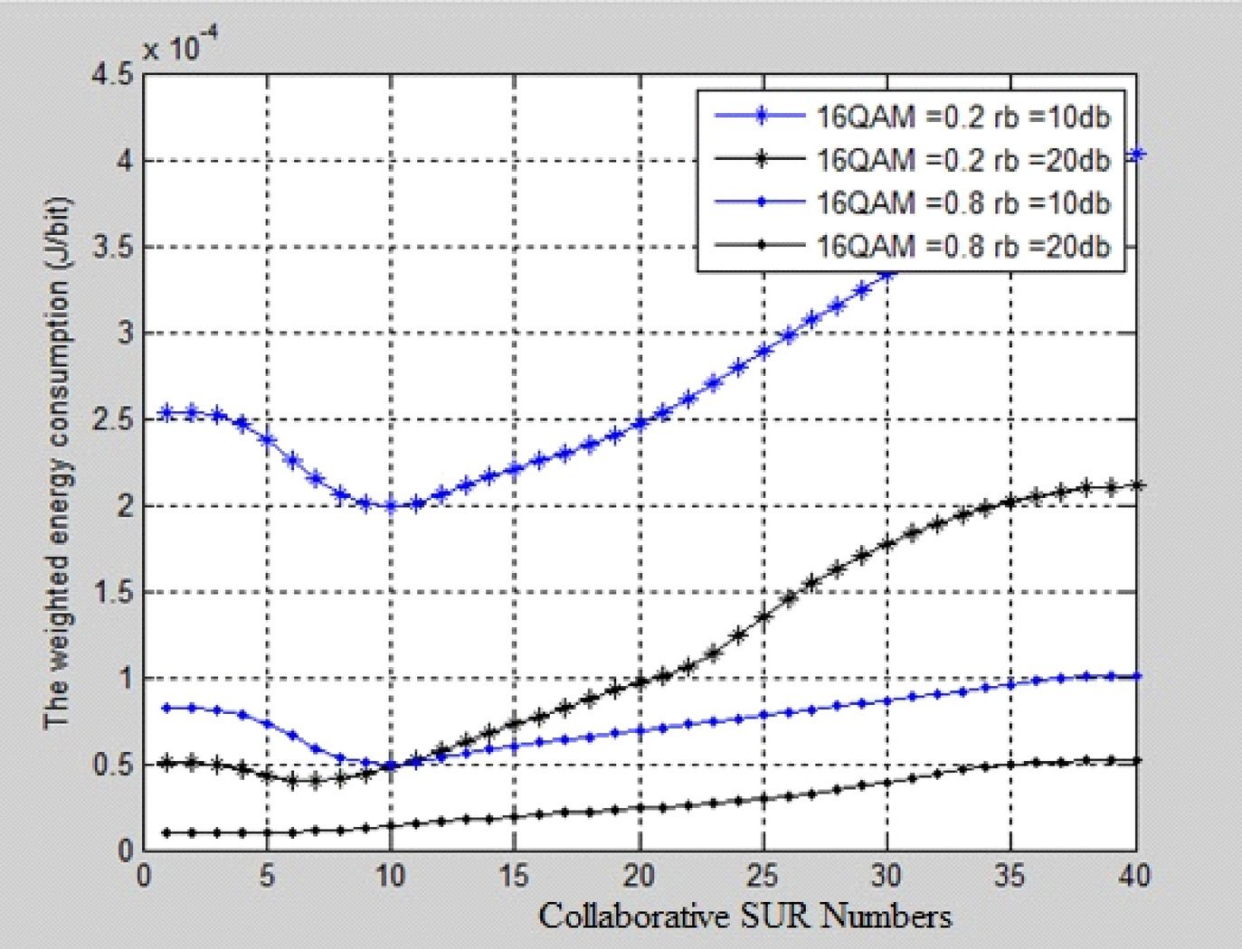
Comparative analysis of weighted energy consumption vs collaborative SUR numbers for 16-QAM.

The collaborative SUR Numbers are taken on the X-axis and weighted Energy consumption in (J/bit) is taken on Y-axis. It has been observed that From Figs. 3, 4, and Table 2, that the optimal SUR numbers are increased with the increasing of the received SNR requirements for a given modulation method, which implies that stricter SNR requirements result in the better transmission link, thus more SURs are required to satisfy secondary transmission BER constraint. For a given modulation method with different sensing overhead, it indicates that sensing overhead influences optimal SUR selection slightly, and the selected optimal SURs are almost the same for different sensing overheads via the EERS scheme. Comparing BPSK, QPSK, and 16QAM, and considering a given sensing overhead, it is realized that fewer SURs are selected in 16QAM than BPSK and QPSK for different SNR requirements, which means that to achieve the minimization of the weighted energy consumption, 16QAM requires fewer SURs than BPSK and QPSK. The aim is that BPSK, QPSK, and 16QAM are all band-limited modulations. A higher modulation order leads to higher bandwidth efficiency. Therefore, for the purpose of energy efficiency, the selected SURs could implement adaptive modulation methods in accordance with SUD received SNR requirements.

**Table 2:** Collaborative SU relay numbers K with various SNR conditions.

Binary PSK	α		Quadrature PSK	α		16-QAM	α	
	0.2	0.8		0.2	0.8		0.2	0.8
ϒ_b_ = 5 dB	3	2.2	ϒ_b_ = 5 dB	4	4	ϒ_b_ = 5 dB	2	2.5
ϒ_b_ = 10 dB	4.5	4	ϒ_b_ = 10 dB	6.5	5	ϒ_b_ = 10 dB	3	3.2
ϒ_b_ = 15 dB	7.1	7.6	ϒ_b_ = 15 dB	9	8	ϒ_b_ = 15 dB	4.7	4.3
ϒ_b_ = 20 dB	11	12	ϒ_b_ = 20 dB	13	14	ϒ_b_ = 20 dB	6.2	5

Figure 4 shows the BER comparison of 16QAM modulation techniques. The collaborative SUR Numbers are taken on X-axis and weighted Energy consumption in (J/bit) is taken on Y-axis. For different SNR conditions, it was found that fewer SU Relays are assigned in 16-QAM than in Binary PSK and Quadrature PSK, indicating that 16-QAM requires fewer SU Relays to achieve a reduction in weighted energy consumption.

**Fig. 5:**
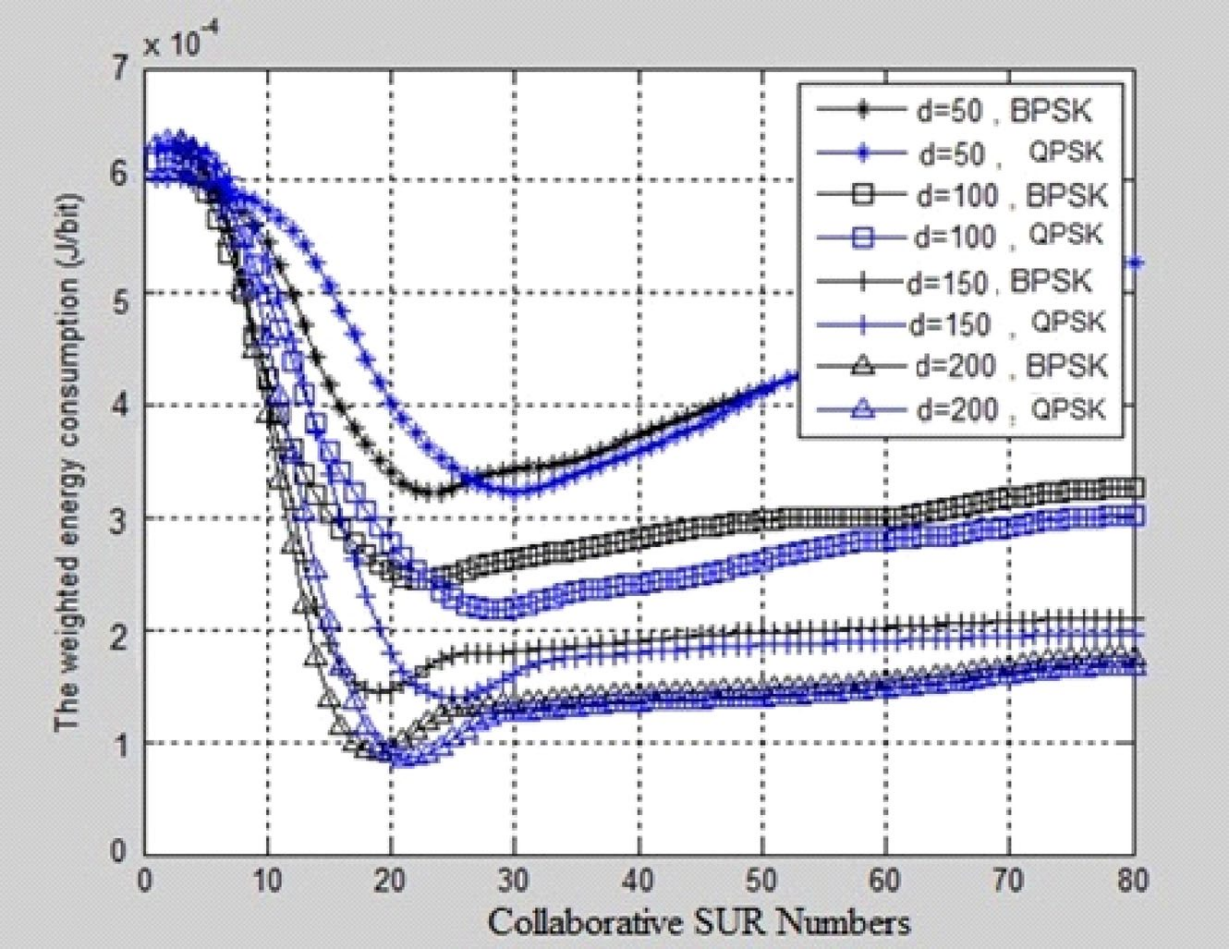
Comparison graph of weighted energy usage based on broadcast distance.

Figure 5 depicts the relationship between collaborative SUR numbers and transmission energy consumption in different transmission distance scenarios. The missing detection probability is taken on the X-axis and weighted Energy consumption in (J/bit) is taken on the Y-axis. A weighted energy consumption comparison for different modulation techniques, including BPSK and QPSK, with the broadcast distance, is shown in Fig. 5. The weighted Energy consumption in (J/bit) and the missing detection probability were plotted on the X–Y axis. The established 20 dB Signal Noise Ratio restriction was experimental. An increased broadcast distance d increases the communication energy usage for identical modulation schemes. When the broadcast distance increases from 50 to 200 m for Binary PSK and Quadrature PSK modulation, it is necessary for 12 more SU Relays to work together to reach the lowest point of transmission energy consumption.

**Fig. 6:**
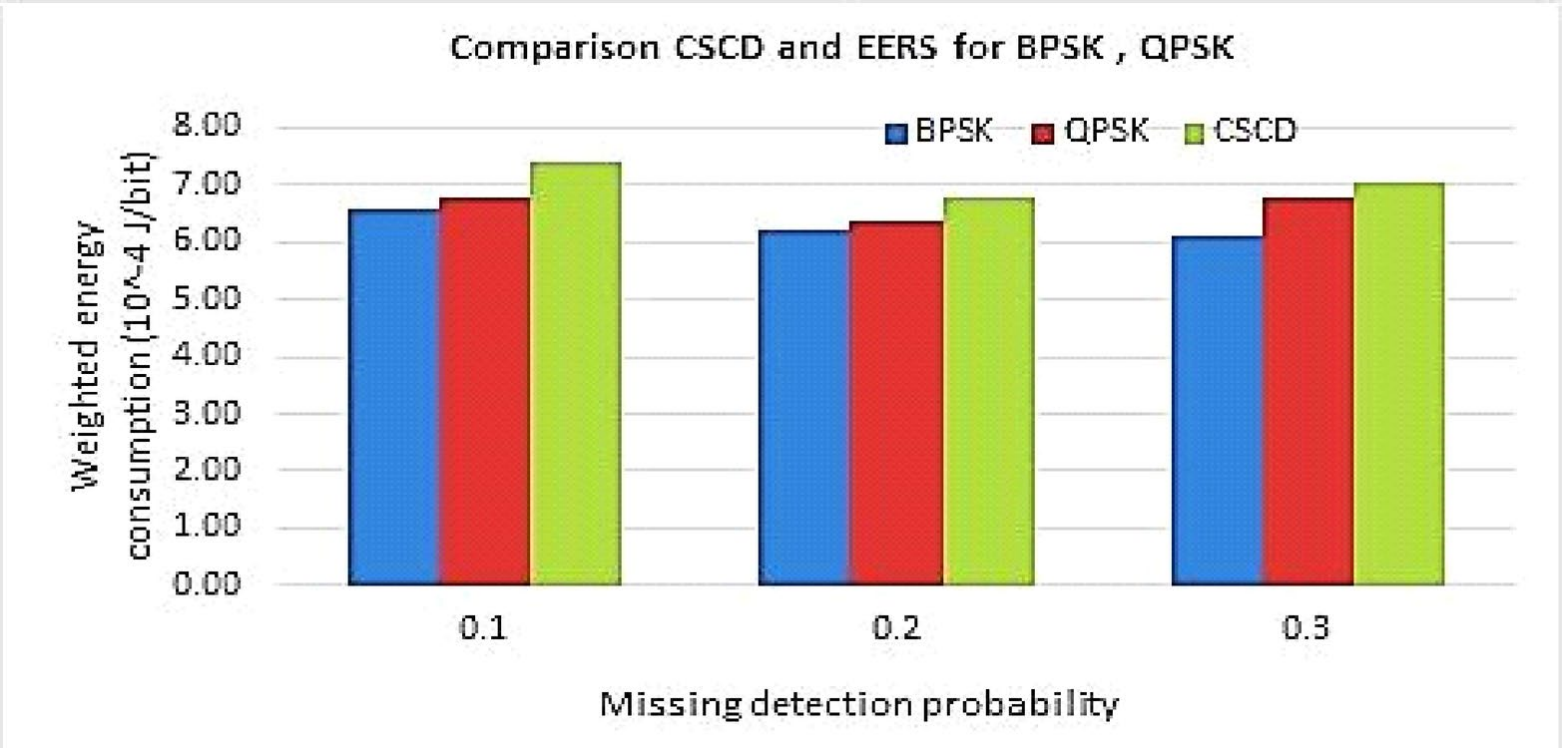
Comparison of the probability of missed detection (MDP) vs weighting method for energy consumption.

The probability of missed detection (MDP) vs. weighting method for the energy consumption charts for the proposed EERS system and the compressed sensing-based collaborative detection (CSCD) system are shown in Fig. 6. The weighted Energy consumption in (J/bit) and the missing detection probability were plotted on the X–Y axis.

**Fig. 7:**
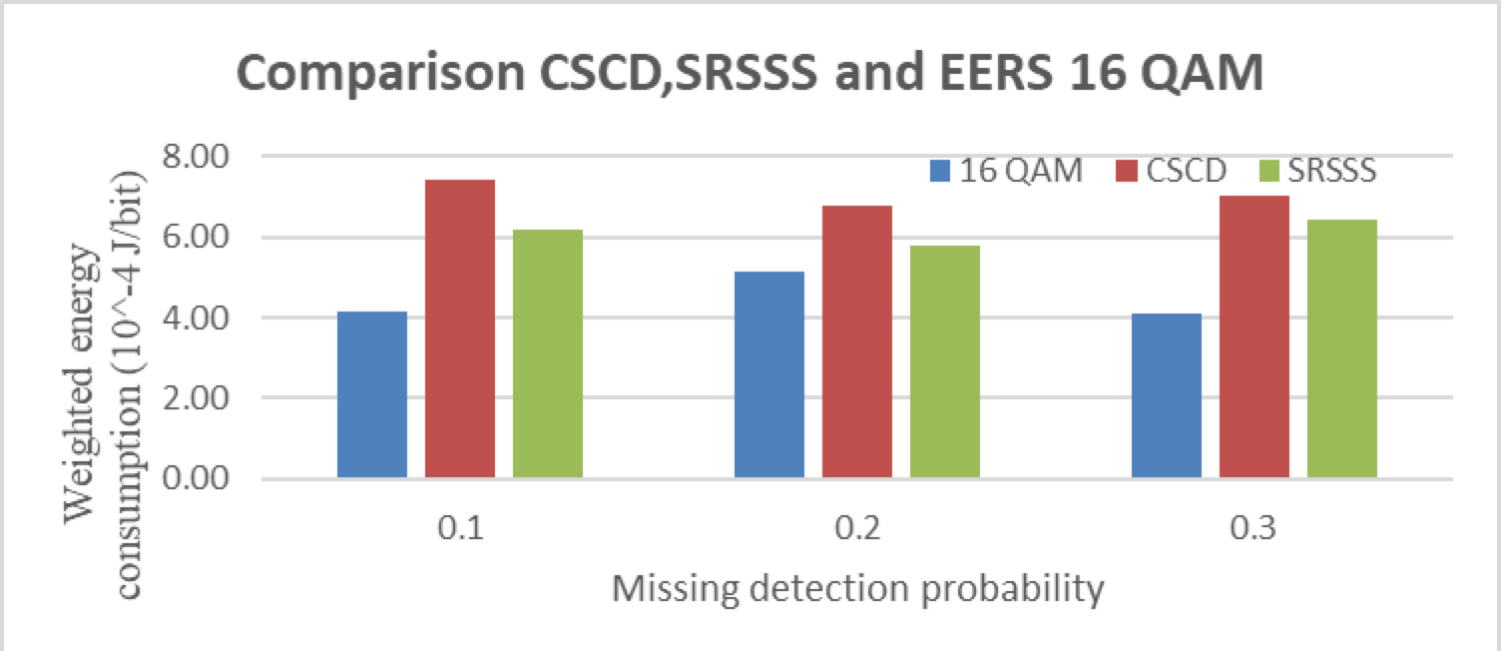
The weighted energy usage for 16-QAM modulation and the missing detection probability (MDP).

The Missing detection probability (MDP) Vs. Weighted energy usage for the 16-QAM modulation is shown in Fig. 7. The weighted Energy consumption in (J/bit) and the missing detection probability were plotted on the X–Y axis. The compressed sensing-based collaborative detection (CSCD), Selective Relay Synthesis Spectrum Sensing (SRSSS), and planned EERS systems are compatible. Compared to the CSCD, SRSSS system, the weighted energy consumption of the EERS system shows substantially lower missing probability values. Additionally, compared with quadrature-PSK and binary-PSK with comparable sensing factors, the EERS method for 16-QAM consumes less energy per bit.

The cooperative SU relay numbers, K, for various SNR conditions are listed in Table 2. According to the observations, 16-QAM requires fewer SU relays than binary PSK and quadrature PSK under various SNR conditions. This means that 16-QAM can reduce the weighted energy consumption with fewer SU relays than the other modulations.

## Conclusion

To increase the energy efficiency of a CRN for 5G communication, an Energy-Efficient Relay Selection (EERS) system was created in this work. MATLAB was used to analyze and evaluate performance metrics such as weighted energy consumption, collaborative SU relay counts, and missing detection probability with Compressed Sensing-based Collaborative Detection (CSCD). The energy consumption of the EERS system is much lower than that of the CSCD system, with similar factors, because it has been observed to offer less weighted energy consumption when compared to the CSCD system in various modulations.

## Data Availability

The data used to support the findings of this study are included in the article. Original data can be provided upon request from S.Esakki Rajavel, Email: rajavel019@gmail.com.
